# Trapping polysulfide on two-dimensional molybdenum disulfide for Li–S batteries through phase selection with optimized binding

**DOI:** 10.3762/bjnano.10.77

**Published:** 2019-03-26

**Authors:** Sha Dong, Xiaoli Sun, Zhiguo Wang

**Affiliations:** 1 School of Electronics Science and Engineering, Center for Public Security Technology Research, University of Electronic Science and Technology of China, Chengdu, 610054, P.R. China

**Keywords:** Li–S batteries, molybdenum disulfide, phase transformation

## Abstract

Introducing anchoring materials into cathodes for Li–S batteries has been demonstrated as an effective way to overcome the shuttle effect and enhance the cycling stability. In this work, the anchoring effects of 2H-MoS_2_ and 1T'-MoS_2_ monolayers for Li–S batteries were investigated by using density functional theory calculations. It was found that the binding energies of Li_2_S*_x_* absorbed on 1T'-MoS_2_ monolayer are in the range of 0.31–2.94 eV, which is much higher than on the 2H-phase. The 1T'-MoS_2_ monolayer shows stronger trapping ability for Li_2_S*_x_* than the 2H-MoS_2_ monolayer. The 1T'-MoS_2_ monolayer can be used as effective anchoring material in cathodes for Li–S batteries.

## Introduction

To satisfy the increasing demand for high-capacity energy storage systems, rechargeable lithium–sulfur (Li–S) batteries have attracted much attention in recent years due to a high theoretical specific energy density of 2567 Wh/kg, a high theoretical capacity of 1672 mAh/g, low cost, non-toxicity, and the abundance of sulfur [1]. The energy density of a Li–S battery is six times higher than that of current commercially used lithium-ion batteries (387 Wh/kg) [2–5]. Typically, a rechargeable Li–S battery is composed of a sulfur cathode and a metallic Li anode, with an organic liquid electrolyte as the ionic conductor, and a porous separator. The Li–S batteries undergo the reaction of 16Li + S_8_ → 8Li_2_S, with a simplified reaction sequence of S_8_ → Li_2_S_8_ → Li_2_S_6_/Li_2_S_4_ → Li_2_S_2_/Li_2_S. Low coulombic efficiency, active material loss, and rapid capacity fading hinder the practical application of Li–S batteries [6]. The insulating nature of sulfur and its lithiation products, Li_2_S_2_ and Li_2_S, leads to low electrical conductivity of the cathode and low rate capability. Dissolved higher-order lithium polysulfides (LPSs) (Li_2_S*_x_*, *x* = 4–8) in the organic electrolyte solvent will migrate and react with the lithium anode, which results in capacity fading and low coulombic efficiency [7–8]. The major issue is the complex diffusion of LPSs, which in combination with the subsequent redox reactions is known as the shuttle effect. The shuttle effect aggravates the cyclic performance of the Li–S battery.

During recent years, many approaches have been devoted to suppressing the shuttle effect and improving the conductivity. Physical confinement of LPSs within host materials with large surface area, such as carbon nanotubes and porous materials, has been a common strategy to minimize the leakage of LPSs. However, the function of physical confinement is limited, and it slows down diffusion for ionic transport [9]. The addition of anchoring materials into the cathodes with a strong binding affinity to LPSs was thought as an efficient approach to improve the electrochemical performance. Due to the polarity of LPSs species, the interaction between LPSs and anchoring materials can be enhanced through polar–polar interactions. Graphene-based materials have been considered as anchoring materials due to their high electrical conductivity [10]. However, the adsorption of polarized LPSs on non-polarized graphene is weak; heteroatom doping is necessary for improving the anchoring effect. Nitrogen doping has been used to modify the anchoring behavior of graphene, and the N-doped graphene showed improved anchoring of Li_2_S*_x_* [11]. Polar materials were explored to trap LPSs, such as metal oxide [12–13] and metal-carbide nanoparticles [14]. Many two-dimensional (2D) materials, such as borophene [15], silicene [16], phosphorene [17], Mxene [18] and MoS_2_ [8], have been investigated as anchoring materials due to their large surface-to-volume ratio. An ideal anchoring material should display a binding energy with LPSs in the range from 0.8 to 2.0 eV in order to effectively trap Li_2_S*_x_* [8], and good electrical and ionic conductivity.

Nanostructured MoS_2_ used as an electrode material for lithium-ion batteries shows a higher specific capacity. Nanoflower MoS_2_/reduced graphene oxides composites exhibited a high specific capacity (1225 mAh/g) and an excellent cycling performance (680 mAh/g) after 250 cycles [19]. MoS_2_ nanoparticles have been used as a starting material for the synthesis of Li–S battery cathodes, since Li_2_S and metallic Mo are formed when MoS_2_ is fully lithiated [20–21]. 2D MoS_2_ (ca. 10 nm thick) can also be used as a protective layer for Li metal anodes to suppress the dendrite formation in Li–S batteries. A threefold improvement in cycle life was shown for protected Li metal compared to bare Li metal, which greatly improved the performance of Li–S batteries [22]. MoS_2_ has been used as anchoring material for LPSs to enhance the performance of Li–S batteries, when it is embedded into a sulfur-rich matrix cathode [23]. However, density functional theory (DFT) calculations showed that the LPSs are weakly bound to 2H-MoS_2_ [8]. The enhanced performance of Li–S batteries using MoS_2_ as anchoring material [23] may be attributed to the strong binding at the edge and terrace sites [24].

2D MoS_2_ shows different polymorphs [25], differing in the position of the S atoms on each side of the Mo atomic layer. 2H-MoS_2_ is the energetically stable phase with semiconductor characteristics, in which the S atoms are located in the lattice positions of a hexagonal close-packed structure. 1T'-MoS_2_ is a meta-stable phase with narrow bandgap, in which each Mo atom is octahedrally coordinated with six S atoms. The phase transformation of 2H→1T' has been widely studied [26–28]. The fundamental mechanisms of this structural transformation are governed by electron transfer [26], so the phase transition can be initiated by treatment with *n*-butyllithium (*n*-BuLi) [27], intercalation of alkali-metal ions [29–30], substitution of Mo by Re atoms [28], electron-beam irradiation [31] and hot-electron injection [32]. Recently, it was reported that MoS_2_/reduced graphene oxide (rGO)/S cathodes for Li–S batteries exhibit outstanding performance. X-ray photoelectron spectroscopy and Raman spectroscopy showed that few-layered MoS_2_ is composed of 1T'-phases and 2H-phases [33]. The composites of 1T'-MoS_2_ with other active materials, such as graphene [34], carbon nanotubes [35], Mxene [36], and SnO_2_ [37], have received much attention regarding the use as cathodes for Li–S batteries. The electrochemical performance including the capacity, rate capability and stability can be greatly improved by using these cathodes.

The phase structure has a profound influence on physical and chemical properties such as electron conductivity and catalytic behavior [38]. The mechanism of 1T MoS_2_ enhancing the electrochemical behavior is not well understood. In this study, we systematically investigated the adsorption of LPSs on 2H-MoS_2_ and 1T'-MoS_2_ monolayers with DFT calculation. Our results show that the 1T'-MoS_2_ monolayer interacts strongly with Li_2_S*_x_*, which will hinder the shuttle effect. Taking into account the better conductivity, 1T'-MoS_2_ monolayers can be used as a conductive anchoring material to design advanced Li–S batteries.

## Results and Discussion

**Figure 1:**
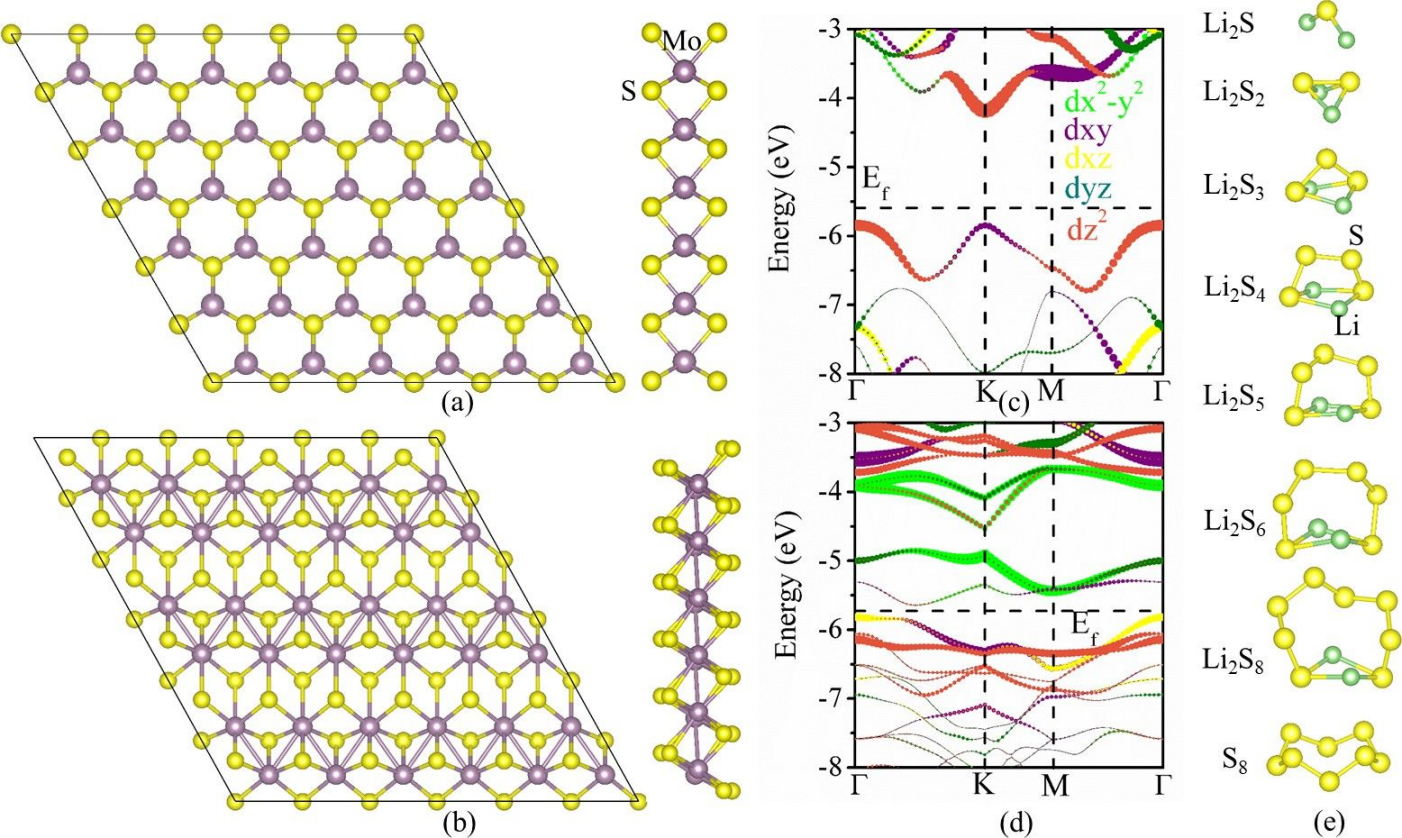
Cross-section and side views of (a) 2H-MoS_2_ and (b) 1T'-MoS_2_ monolayers. Orbital-decomposed band structures along high-symmetry points of (c) 2H-MoS_2_ and (d) 1T'-MoS_2_ monolayers. (e) The optimized atomic configurations of Li_2_S*_x_* (*x* = 1–8) and S_8_ in their ground states.

Both 2H-MoS_2_ and 1T'-MoS_2_ monolayers exhibit a structure with three atomic layers, in which the Mo atomic layer is sandwiched by two S atomic layers. The 6 × 6 supercells for 2H-MoS_2_ and 1T'-MoS_2_ monolayers used in the work are shown in Figure 1a and Figure 1b, respectively. The electronic band structures along high-symmetry points are shown in Figure 1c and Figure 1d, respectively. The 2H-MoS_2_ monolayer is a semiconductor with a direct bandgap of 1.67 eV, both the conduction band minimum (CBM) and valence band maximum (VBM) are located at the K point, which is consistent with previous DFT calculations [39]. 1T'-MoS_2_ is a narrow-bandgap semiconductor with a bandgap of 0.15 eV.

Various intermediates, Li_2_S*_x_* (*x* = 1–8), of LPSs were observed in Li–S batteries [40]. The optimized atomic configurations of LPSs in the ground state are shown in Figure 1e. All Li_2_S*_x_* compounds exhibit a nonplanar shape instead of sulfur chains with terminal Li atoms. The shortest bond length of Li–S increases with the increase of *x* for *x* < 5. For higher *x*, there is no linear dependence. The bond -length values are 2.082, 2.213, 2.307, 2.333, 2.359, 2.354, and 2.330 Å for Li_2_S, Li_2_S_2_, Li_2_S_3_, Li_2_S_4_, Li_2_S_5_, Li_2_S_6_, and Li_2_S_8_, respectively. Li_2_S and Li_2_S_2_ have *C*_2_*_v_* symmetry, Li_2_S*_x_* (*x* = 3–8) has *C*_2_ symmetry, and S_8_ with a puckered ring structure has a *D*_4_*_d_* symmetry.

The trapping of Li_2_S*_x_* on 2H-MoS_2_ and 1T'-MoS_2_ monolayers was evaluated by the calculation of the binding energy (*E*_b_) with Equation 1

**Figure 2:**
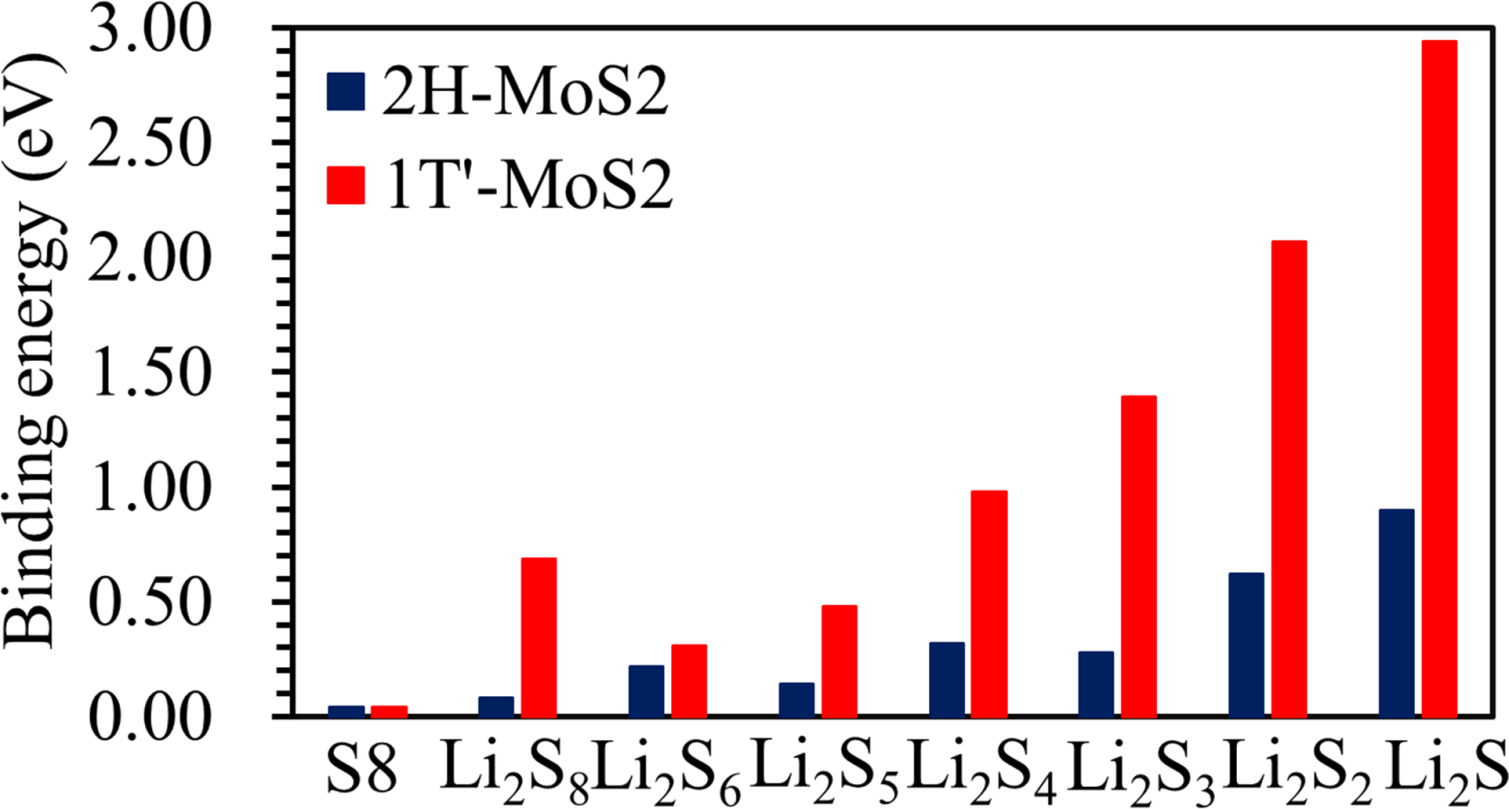
The calculated binding energies of Li_2_S*_x_* on 2H-MoS_2_ and 1T'-MoS_2_ monolayers.

[1] $$E_{\text{b}}=E_{{\text{Li}}_{\text{2}}{\text{S}}_{x}{\text{-MoS}}_{\text{2}}}-E_{{\text{MoS}}_{\text{2}}}-E_{{\text{Li}}_{\text{2}}{\text{S}}_{x}},$$

where 
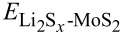
 and 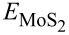 are the total energy of the MoS_2_ monolayer with and without Li_2_S*_x_* adsorption. 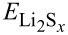 is the energy of an isolated Li_2_S*_x_* molecule in a cubic lattice with a cell length of 30 Å. A more negative binding energy indicates a stronger adsorption interaction between the MoS_2_ monolayer and the Li_2_S*_x_* molecule. The calculated binding energy is shown in Figure 2. The binding energy of Li_2_S*_x_* absorbed on a 2H-MoS_2_ monolayer decreases from 0.90 to 0.08 eV as *x* increases from 1 to 8. For Li_2_S*_x_* absorbed on a 1T'-MoS_2_ monolayer, the binding energy decreases from 2.94 to 0.64 eV. The 1T'-MoS_2_ monolayer shows stronger trapping ability for Li_2_S*_x_* than the 2H-MoS_2_ monolayer. The orbital-decomposed band structures of 2H-MoS_2_ and 1T'-MoS_2_ monolayers are shown in Figure 1c and Figure 1d, respectively. The unoccupied lowest conduction band of the 2H-MoS_2_ monolayer at the K point is dominated by the 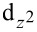 orbital, whereas that of 1T'-MoS_2_ monolayer is dominated by the d*_xy_* and 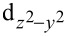 orbitals. As the Li_2_S*_x_* absorbed on the monolayer, electron transfers from Li_2_S*_x_* to the unoccupied lowest states of the monolayer. The d*_xy_* and 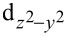 orbitals of the 1T'-MoS_2_ monolayer are lower in energy than the 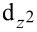 orbital of 2H-MoS_2_, thus leading to a stronger binding. Both 2H-MoS_2_ and 1T'-MoS_2_ monolayers show less trapping of S_8_ with a binding energy of 0.04 eV. The binding energies of Li_2_S*_x_* on borophene are in the range from −1.00 to −3.00 eV [15] and on Ni-doped graphene are in the range from 1.13 to 1.40 eV [11]. The 1T'-MoS_2_ monolayer can be used as a conductive anchoring material to design advanced Li–S batteries.

Detailed analysis of the atomic structure shows that the Li atom of Li_2_S*_x_* is energetically favorable to bind with the 2H-MoS_2_ monolayer. The Li atom prefers to locate at the top position above the Mo atom, which is similar to the Li adsorption on 2H-MoS_2_ monolayers [41]. The distance between the Li atom and the plane of S atoms increases from 2.027 to 3.511 Å as *x* increases from 1 to 8 in Li_2_S*_x_*. The increased distance results in the weak trapping of Li_2_S*_x_* by the 2H-MoS_2_ monolayer. As the LPSs are absorbed on the 1T'-MoS_2_ monolayer, except for Li absorbed on the S plane, one of the S atoms in Li_2_S*_x_* (*x* = 1–3) is also bound to the S plane, thus increasing the anchoring behavior. The distance between the Li atoms in Li_2_S*_x_* and the plane of S atoms of the 1T'-MoS_2_ monolayer is much shorter than that to the 2H-MoS_2_ monolayer. Hence, the 1T'-MoS_2_ monolayer exhibits a stronger trapping of Li_2_S*_x_* than the 2H-MoS_2_ monolayer. The puckered ring structure S_8_ prefers to align parallel to the surface of both 2H-MoS_2_ and 1T'-MoS_2_ monolayer with distances of 3.99 and 3.67 Å, respectively. The large distance and the small binding strength of S_8_ on 2H-MoS_2_ and 1T'-MoS_2_ monolayers indicate that the interaction mainly originates from van der Waals interactions.

**Figure 3:**
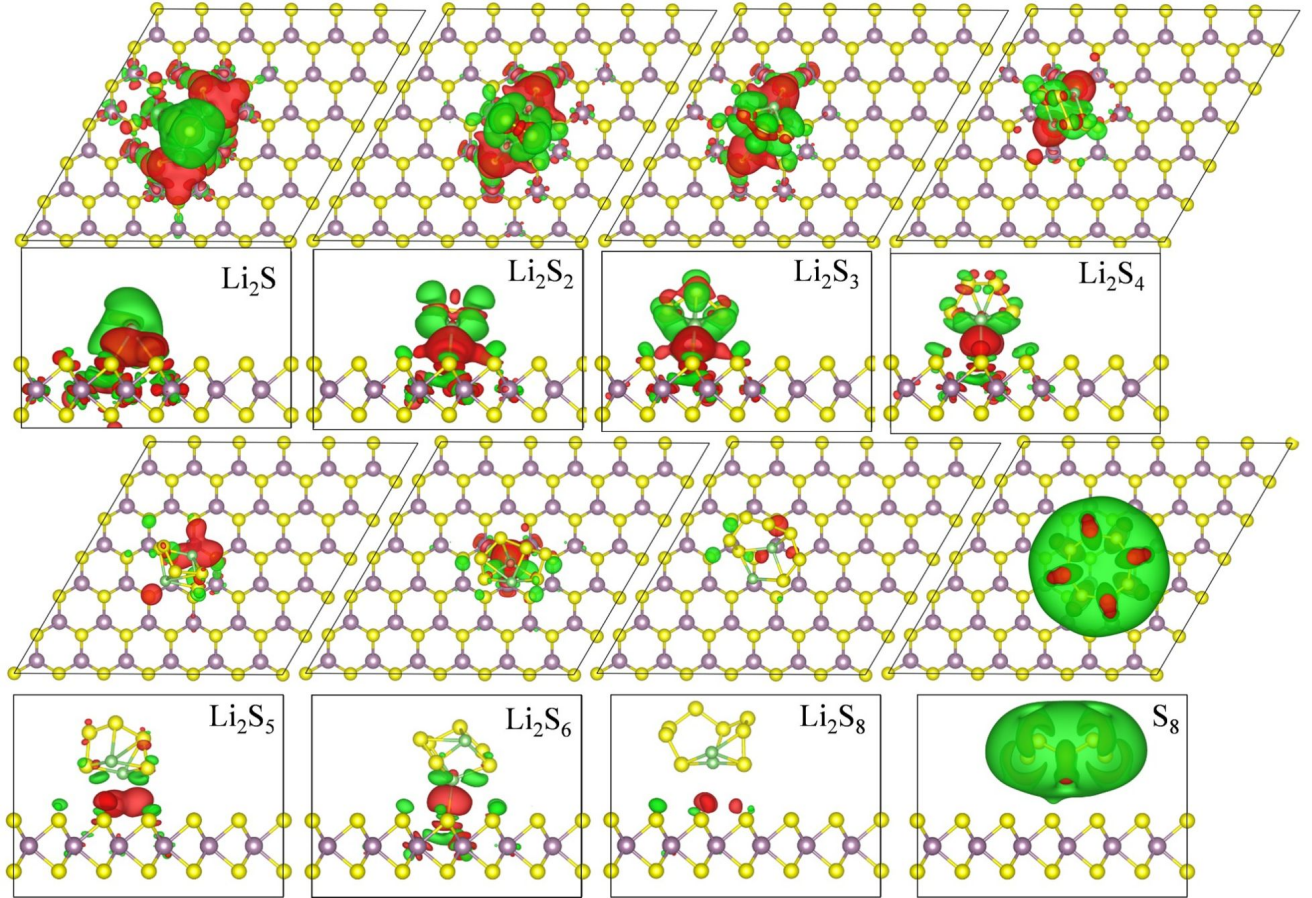
Isosurface (0.0005 e/Å^3^) of the charge distributions of Li_2_S*_x_* absorbed on 2H-MoS_2_ monolayer. The red and green surfaces correspond to charge gain and loss, respectively.

**Figure 4:**
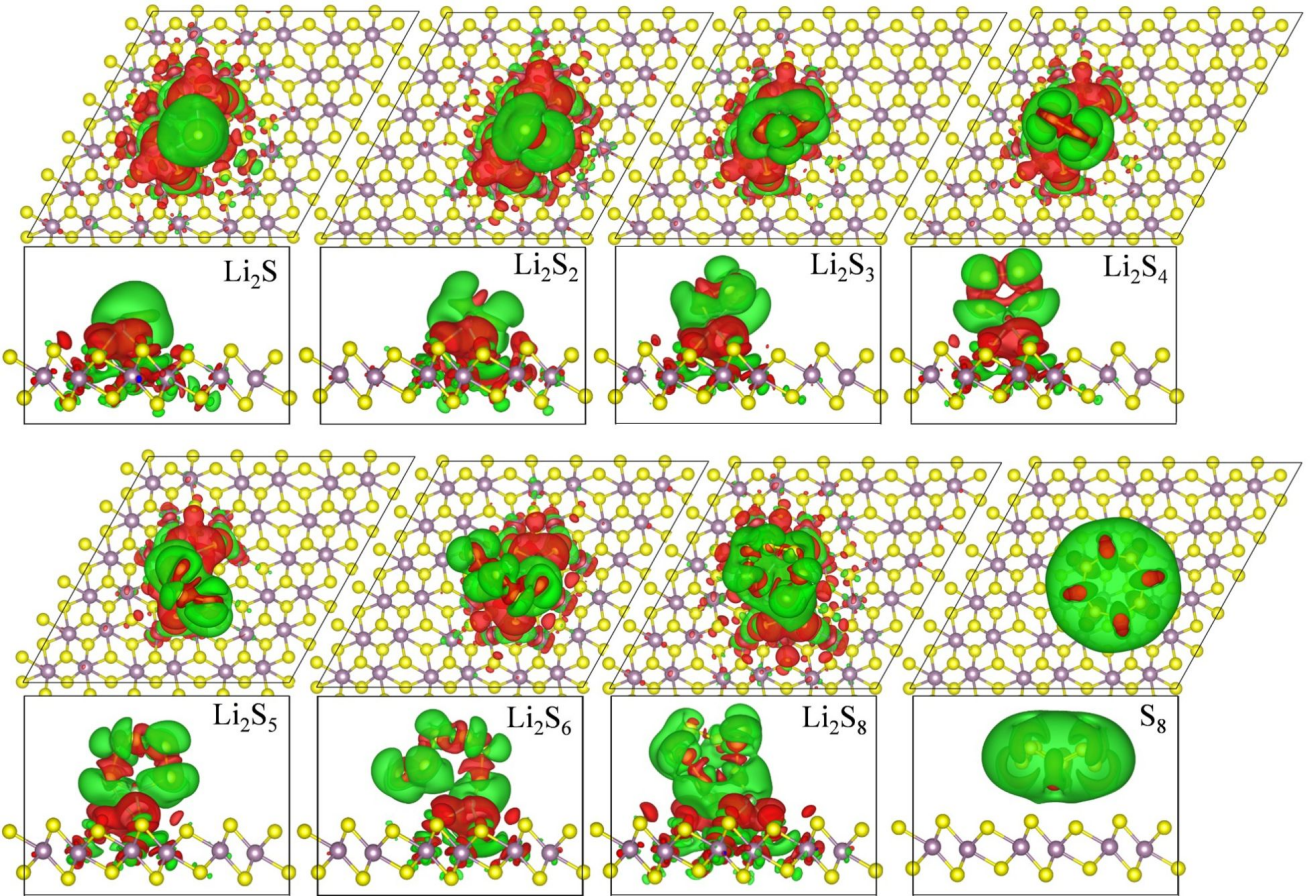
Isosurface (0.0005 e/Å^3^) of the charge distributions of Li_2_S*_x_* absorbed on 1T'-MoS_2_ monolayer. The red and green surfaces correspond to charge gain and loss, respectively.

To understand the binding between LPSs and the 2HMoS_2_- and 1T'-MoS_2_ monolayers, the charge-density difference was calculated using Equation 2:

[2] $$\Delta \rho =\rho_{{\text{Li}}_{\text{2}}{\text{S}}_{x}{\text{-MoS}}_{\text{2}}}-\rho_{{\text{MoS}}_{\text{2}}}-\rho_{{\text{Li}}_{\text{2}}{\text{S}}_{x}},$$

where 
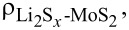
 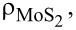 and 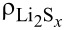 are the charge densities of the adsorbed system, the MoS_2_ monolayer, and the Li_2_S*_x_* molecule, respectively. The differences of charge densities for Li_2_S*_x_* absorbed on a 2H-MoS_2_ monolayer are shown in Figure 3. The red and green surfaces correspond to charge gain and loss, respectively. It can be observed that there is charge depletion in Li_2_S*_x_* and the 2H-MoS_2_ monolayer, and that charge accumulates between the Li atoms and the S atoms of 2H-MoS_2_, suggesting a chemical Li–S bond between Li_2_S*_x_* and the 2H-MoS_2_ monolayer. With increasing *x* the charge redistribution becomes less pronounced, and there is almost no charge exchange between Li_2_S_8_ and the 2H-MoS_2_ monolayer, causing only weak adsorption. This agrees with the fact that the binding energy of Li_2_S*_x_* absorbed on the 2H-MoS_2_ monolayer decreases as *x* increases from 1 to 8. The charge-density differences for Li_2_S*_x_* absorbed on a 1T'-MoS_2_ monolayer are shown in Figure 4. In contrast to the case of Li_2_S*_x_* absorbed on the 2H-MoS_2_ monolayer, the charge redistribution is more apparent, indicating the strong trapping ability for Li_2_S*_x_*. For both 2H-MoS_2_ and 1T'-MoS_2_ monolayers, there is no charge migration between the monolayer and the puckered ring structure S_8_, which is consistent with the corresponding small binding energies.

The working mechanism of Li–S batteries includes the following steps, octasulfur is reduced to long chains of LPSs, Li_2_S*_x_* (6 < *x* ≤ 8), and further to lower-order LPSs, Li_2_S_x_ (2 < *x* ≤ 6), the final product of Li_2_S is formed upon discharging; and the charging process occurs through the reverse reactions [40]. The insulating nature of sulfur and the lithiation products (Li_2_S_2_ and Li_2_S), and the dissolution of higher-order Li_2_S_x_ (*x* = 4–8) are the main challenges in the application of Li–S batteries [7–8]. An ideal anchoring material with binding energies with LPSs in the range from 0.8 to 2.0 eV [15], and with good electrical and ionic conductivity is desirable for improving the electrochemical performance of Li–S batteries. Although the 2H-MoS_2_ monolayer shows good Li conductivity, it is a semiconductor with weak binding energies for LiPSs. Through phase engineering of the 2H-MoS_2_ to the 1T'-MoS_2_ monolayer, the anchoring behavior can be greatly improved. 1T'-MoS_2_ also exhibits good electrical and ionic conductivity. Hence, the electrochemical performance of Li–S batteries is improved by using 1T'-MoS_2_ as additive in the cathodes [33–37].

## Conclusion

In conclusion, the anchoring effects of 2H-MoS_2_ and 1T'-MoS_2_ monolayers for Li–S batteries were investigated by using DFT calculations. It was found that the binding energies of Li_2_S*_x_* absorbed on the 1T'-MoS_2_ monolayer are in the range of 0.31–2.94 eV, whereas they are in the range of 0.08–0.90 eV for Li_2_S*_x_* absorbed on the 2H-MoS_2_ monolayer. The 1T'-MoS_2_ monolayer shows a stronger trapping of Li_2_S*_x_* than the 2H-MoS_2_ monolayer. The 1T'-MoS_2_ monolayer can be employed as effective anchoring material in cathodes for Li–S batteries.

## Simulation Details

The same simulation method and models of [26] were used in the present work. All spin-polarized DFT calculations were performed with the Vienna ab initio simulation package (VASP) [42] plane-wave simulations. Electron–ion interaction and electron exchange–correlation were described using the projector augmented wave (PAW) method [43] and the generalized gradient approximation (GGA) was described using the Perdew–Burke–Ernzerhof (PBE) function, respectively. The energy cutoff for the plane-wave basis expansion was chosen to be 520 eV. To avoid the interaction between the periodic images, a 6 × 6 supercell of a MoS_2_ monolayer, which contains 36 Mo and 72 S atoms, was used to investigate the adsorption of Li_2_S*_x_*. A 25 Å vacuum space was constructed perpendicular to the monolayers. The Brillouin zone was integrated using the Monkhorst–Pack scheme [44] with a 3 × 3 × 1 k-grid for the geometry optimization. All atomic positions and cell parameters were relaxed until the force on each atom is less than 0.02 eV/Å.
